# Can the Functional Movement Screen Method Identify Previously Injured Wushu Athletes?

**DOI:** 10.3390/ijerph18020721

**Published:** 2021-01-15

**Authors:** Di Wang, Xiao-Mei Lin, Juha-Pekka Kulmala, Arto J. Pesola, Ying Gao

**Affiliations:** 1 Department of Public Physical and Art Education, Zhejiang University, Hangzhou 310058, China; dwang@zju.edu.cn; 2 Department of Sports Science, College of Education, Zhejiang University, Hangzhou 310058, China; Linxm66@163.com; 3 Motion Analysis Laboratory, Children’s Hospital, University of Helsinki and Helsinki University Hospital, FI-00290 Helsinki, Finland; juha-pekka.kulmala@hus.fi; 4 Active Life Lab, South-Eastern Finland University of Applied Sciences, FI-50100 Mikkeli, Finland; Arto.Pesola@xamk.fi

**Keywords:** wushu sport, elite athletes, sport injury, functional movement screen

## Abstract

The functional movement screen (FMS) is commonly used to evaluate sports injury risks, but no study has been reported for Wushu athletes. The aim of this study was to identify optimal FMS cut-off points for previously injured Wushu athletes and to examine the associations with other possible factors. In this study, a total of 84 Chinese Wushu athletes (15.1 ± 4.5 years old, 51% male) with a minimum of two years of professional training background in either Taiji, Changquan, or Nanquan were assessed by the FMS. Video recordings were used to confirm the scoring criteria, and previous injuries were assessed based on face-to-face interviews. An optimal cut-off of the FMS score was investigated by receiver operating characteristic curves with sensitivity and specificity. We found that FMS score of less than 16 (sensitivity = 80%, specificity = 56%) was related to an increased occurrence of injuries (odds ratio = 5.096, 95%CI: 1.679–15.465) for the current study sample. The training type and training levels were related with FMS scores. More than half of the athletes (58%) had FMS asymmetry and 21% of athletes reported pain while performing the FMS protocol. Future prospective studies are recommended to use FMS with cut-off of 16 points in Wushu athletes.

## 1. Introduction

Wushu is a traditional sport in China. Nowadays Wushu is practiced over the world and can be better known from one of its sub-types, Taiji. Practicing Taiji has been shown to provide some therapeutic benefits, such as improved quality of life and physical functions [1]. Other types of Wushu include Changquan and Nanquan which are also known to benefit physical fitness and health [2]. Regular practice of the movement patterns can lead to improvements in balance and stamina [2]. Competitive Wushu routine is a traditional sport, where the movement difficulty and beauty are subjectively scored by a judge. The Wushu routine movements are mainly based on jumping, turning, and single-leg stance, with strict requirements on movement stability. Along with the Olympic tenet of “higher, faster and stronger”, Wushu routine competition is becoming more and more intense. High injury risks have been reported for professional Wushu athletes, in particular regarding serious non-contact injuries in the knee, ankle, and back [3,4]. Such high injury risks call for combining evidence-based training and evaluation methods to better prevent sport-related injuries in the large Wushu athlete and amateur base.

The functional movement screen (FMS) was developed to assess an individual’s ability to perform fundamental movements [5,6]. The FMS is comprised of seven functional tests: deep squat, hurdle step, in-line lunge, shoulder mobility, active leg raise, trunk stability push-up and rotary stability [7]. Each of these tests is scored on a scale from zero to three and those scores are summed to a composite score that can range from zero to twenty-one [7]. The underlying idea of the FMS is that individuals need to be able to properly perform these fundamental movements before they can focus on improving power, strength, and flexibility. Since its development, an increasing number of studies have utilized the FMS to evaluate and predict the risk of sports injuries in different exercise regimens [8,9]. The FMS has been shown an acceptable intra-rater and test-retest reliability [10,11,12].

The FMS has been commonly used to identify individuals who may be at a high risk for developing injuries [13]. For instance, the seminal study of Kiesel et al. found that professional football players, who received the FMS score of 14 or less, were at a higher risk (odds ratio was 11.67) of serious injuries during the follow-up season [14]. However, while many FMS studies have used the cut-off point of 14 for identifying those at high risk of injury, it has been suggested that a higher composite score (cut-off point >14) may be more suitable for predicting injury risks at the high-level athletes [15]. Furthermore, several controversies exist regarding the predictive validity of the FMS and its underlying factors, e.g., sport type, which may be related to elevate injury risk [16,17,18]. More evidence is needed to identify the utility of FMS in professional athletes and its associations for those are at high risk of injury and high motor skill level [19]. To date, no studies have utilized FMS in Wushu routine sports, even though Wushu athletes are at high injury risk. Thus, it is necessary to assess the application of FMS and its relationship with the injury on this specific profession.

The aim of this study was to identify optimal FMS cut-off points for identifying previously injured Wushu athletes, as well as to examine the association between the FMS scores and sport-related injury history in different levels of Wushu athletes. Furthermore, we examined if the training level and training years are associated with low FMS scores.

## 2. Methods

### 2.1. Participants

A total of eighty-four Wushu athletes participated in the study. They were recruited from professional Wushu sports teams from Zhejiang Province team (*n* = 34) and Chen sports school (*n* = 50) in Hangzhou city, China. The range of age was 8–25 years old and all had at a minimum of two years professional training history. There are three different types of Wushu routine sports included in the current study: Taiji, Changquan, and Nanquan. For the professional levels, there are four certified levels including the national level (Top), the first-class (Q1), the second-class (Q2), and the third-class (Q3). The study was approved by institutional review board of Zhejiang University (2018.6.7). All participants and their legal guardians if under age 18 were informed of the risks and benefits of participation in the study before signing the written informed consent. The study was conducted in agreement with the Declaration of Helsinki.

### 2.2. Study Design and Procedures

This study was a cross-sectional study design. Data collection was carried out during a single session including interview and FMS assessment. The participants self-reported their demographic characteristics during a face-to-face interview session, including age, gender, height (cm), body mass (kg), professional levels, and athletic participation for training years and types. The height and body mass were consistent with their recent physical examination. The injury history was screened according to anatomical locations, including the knee, shoulder, ankle, wrist, and back. Information regarding healthcare needs following a given injury was collected. All injury history was documented in their team medical system, which was recorded for their treatment and recover situation. All included participants were in pre-season practice and/or conditioning programs, and were required to have no current injuries, with a minimum of 3–6 months from the previous injury treatment cessation. The non-contact musculoskeletal injuries were defined by the following criteria: (1) the injury occurred as a result of participation in Wushu practice and/or conditioning programs; (2) the injury required medical care or certified physician care and they had prohibited full participation in pre-season practice and/or conditioning programs. The injury history was extracted from the recent two years injury recordings. Following the interview session, the FMS was assessed in a rolling recruitment for each test. Before the FMS, all participants spent at least 30 minutes under quiescent condition and between each test they were resting for at least for 15 minutes.

### 2.3. The FMS Protocol

The FMS assessment was performed by a qualified physiotherapist, a physical fitness coach, and an accredited exercise physiologist, who had either obtained the FMS certification at least for 2 years or a physician qualification at least for 6 years. Participants were instructed following the standard FMS™ protocol and completed 7 different tests in order of the deep squat, hurdle step, in-line lunge, shoulder mobility, active straight leg raise, trunk stability push-up and rotary stability test and three clearing tests [5,6]. The standard scoring criteria were outlined on an ordinal scale from 0 to 3 by the administrators. All administrators were responsible for the same test to reduce the potential for inter- scoring discrepancies. Although FMS method has been reported to have a good reliability and intra-class correlation coefficient ≥0.75 [20], risk for difference between administrators was minimized by standardized FMS procedures. The scores for the 7 tests were 0, 1, 2, or 3. A score of 3 was given if the test was performed without compensations, a 2 if compensations occurred during the test, a 1 if the individual was unable to get into the test position or to complete the test, and a 0 was recorded if any pain was perceived during the test [5,6]. Furthermore, participants completed three clearing tests to rule out painful movement for the shoulder mobility, trunk stability push-up, and rotary stability tests. If the perceived pain was reported, then the scoring for the specific test was set for 0 accordingly. The perceived pain was reported based on all 7 tests and three clearing tests, which was confirmed with the physiotherapists. Five of the tests including hurdle step, in-line lunge, shoulder mobility, active straight leg raise, and rotary stability were performed bilaterally to assess asymmetry. The asymmetry was defined when the differences existed between the participant’s left and right scores, and then the lower of the two was utilized in computing the composite score accordingly for each bilateral test. The presence of asymmetries was also recorded for these five tests. The final sum of scores from 7 tests was calculated for a total score, with a higher score indicating a better performance. All tests were recorded by video to confirm the scoring criteria.

### 2.4. Statistical Analyses

Participant characteristics were described using means and standard deviations (SD) and their 95% confidence intervals (CI) or numbers with percentages. Statistical analyses were conducted using IBM SPSS for Windows Version 25.0 (IBM Corp., Armonk, NY, USA). A probability level of *p* < 0.05 (two-tailed) was considered statistically significant. The injury risk was defined by dichotomy for injury history (set at 0) and without injury history (set at 1). The non-normality distribution was reported for the FMS scores. A Mann-Whitney Test was used to compare gender differences in the FMS total scores, asymmetries, and injury risks. Since there were no significant differences between genders, data were pooled for further analyses.

The Mann–Whitney Test was performed to compare FMS scores between those injured and those who were not injured, as well as between symmetric versus asymmetric athletes. Receiver operating characteristics (ROC) curves were used to investigate the optimal cut-offs for FMS scores to discriminate between those injured and those who were not injured. The area under the curve (AUC) with their 95% CI is considered a measure of the utility of the predictor variable and represents the trade-off between the correct identification of participants with injuries (sensitivity) and the correct identification of participants without injuries (specificity). The cut-off that maximized the value of the square root of the sum of the sensitivity squared and specificity squared is reported. An AUC of 1 represents the ability to perfectly identify with injuries from without injuries, whereas an AUC of 0.5 indicates no greater predictive ability than chance alone [21]. Once the cut-off score was identified, a 2 × 2 contingency table was created dichotomizing those who had an injury and those who did not, and those above and below the cut-off score on the FMS. A Fisher’s exact test was performed and odds ratios (OR) with 95% CI were then calculated.

Furthermore, a Spearman correlation (r) was used to examine the relations between the training years and FMS scores. Further adjustment for the ages was examined by using a partial correlation. The strength of correlation was interpreted as weak (<0.19), low (0.20–0.39), moderate (0.40–0.59), strong (0.60–0.79), or very strong (>0.80) [22]. Kruskal–Wallis test was used to test different training levels and training types for the FMS scores. If it revealed significant effects of training levels or training types, least significant difference post-hoc multiple comparisons were used to identify the difference.

## 3. Results

**Figure 1:**
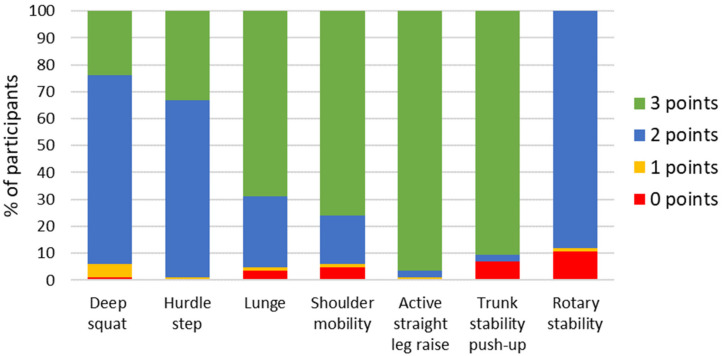
The frequency of FMS scores across the 7 tests (0–3 points).

**Figure 2:**
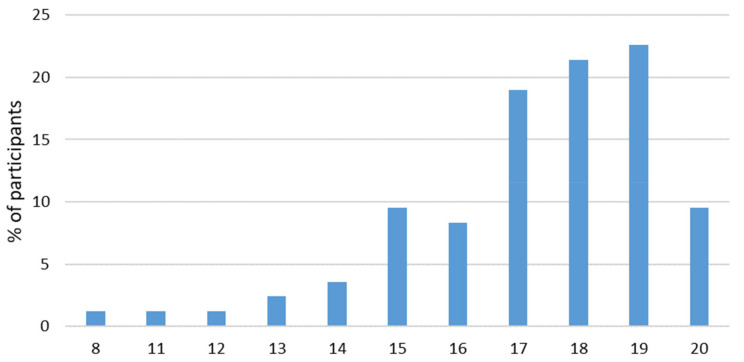
The frequency of FMS scores in total (8–21 points).

A total of 84 Wushu athletes (49% female) aged 8–25 were included in this study, and of them, 21.4% (*n* = 18) had injury history. Furthermore, 58.3% of participants (*n* = 49) had asymmetry and 21.4% of them (*n* = 18) perceived pain during the FMS tests and clearing tests. Participants’ characteristics, training years, types, and levels are reported in Table 1. The mean training years among different athletes’ levels was 10.6 ± 3.2 y for Top, 7.6 ± 2.3 y for Q1, 5.2 ± 2.5 y for Q2 and 2.6 ± 1.0 y for Q3. The FMS composite scores were in a range of 8 points to 20 points. The detailed FMS scores for each test and total FMS scores in all participants are shown in Table 2 and their frequency for each test and total FMS scores are shown in Figure 1 and Figure 2. Different training levels and training types are presented in Supplementary Table S1 as well as Supplementary Figures S1 and S2.

Compared with those who had not been previously injured, those with injury history had lower FMS scores (17.7 ± 1.6 vs. 15.5 ± 3.1, *p* = 0.005). Compared with those who were symmetric, those with asymmetries had lower FMS scores (18.5 ± 1.5 vs. 16.4 ± 2.2, *p* < 0.001).

**Figure 3:**
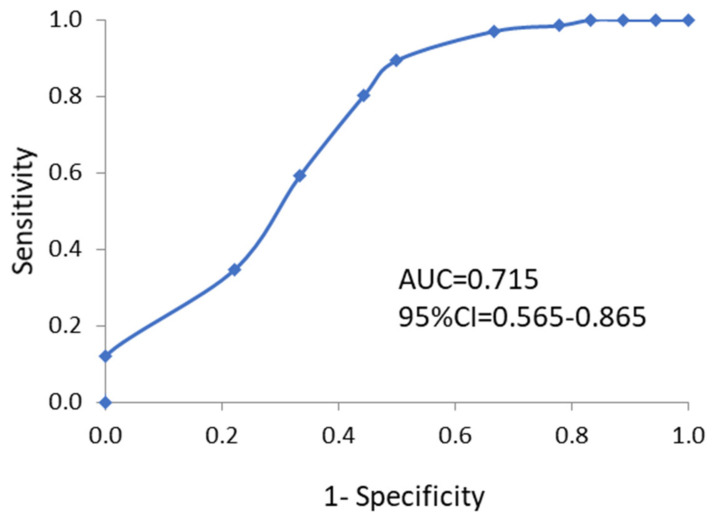
Receiver operating characteristics (ROC) curves for FMS scores and injury risk. The area under the curve (AUC) with 95% confidence interval (CI) was determined.

The AUC with the 95%CI is shown in Figure 3. The optimal cut-off for discriminating previously injured versus uninjured athletes was 16, where the sensitivity was 80.3% and specificity was 55.6%. When applied with the cut-off score of 16, a 2 × 2 contingency table is present (Table 3). Those with an FMS score of ≤16 was found to be significantly more likely to have an injury (Fisher’s exact test, *p* = 0.006), where OR was 5.096 (95%CI: 1.679–15.465).

Furthermore, a moderate negative correlation between the training years and FMS scores was found (r = −0.407, *p* < 0.001). However, after adjusting for age, the strength of this correlation was low (r = −0.207, *p* = 0.030, one-tailed). Non-parametric tests revealed the effects of training levels for FMS scores (*p* = 0.049), where the Top athletes had a lower score than level Q2 (16.1 ± 2.9 vs. 17.9 ± 1.8, *p* = 0.002) and Q3 (16.1 ± 2.9 vs. 17.5 ± 1.3, *p* = 0.026), but not for Q1 (16.1 ± 2.9 vs. 17.0 ± 2.2, *p* > 0.05). There was a significant difference between training types of Taiji, Nanquan and Changquan (*p* = 0.030), Taiji group had a higher scoring than Nanquan (18.3 ± 1.4 vs. 16.8 ± 1.7, *p* = 0.040) and Chanquan (18.3 ± 1.4 vs. 17.0 ± 2.5, *p* = 0.047).

## 4. Discussion

As hypothesized, the FMS scores were relatively high in Wushu athletes, with 73% scoring above 16 points. The results of this study showed that the FMS with cut-off score of 16 provided an acceptable sensitivity and specificity for differentiating the previously injured athletes from those without injury history. These retrospective results are the first published application of the FMS method in Wushu sports and should be confirmed in prospective designs in order to evaluate whether FMS can be used to predict future injury risks in this athlete group.

The underlying theory behind the FMS is that an individual needs to be able to properly perform these fundamental movements before they can focus on improving power, strength, and flexibility. Wushu routine includes many difficult actions, and requires players to have physical flexibility and joint agility to complete Wushu routine movements [5,6]. Many studies have used the FMS method to evaluate and demonstrate its effectiveness in professional athletes in different sports, but less research has been done for the Wushu routine sports, especially in high level athletes. In the current study, we used FMS in three different types of Wushu routine athletes including Taiji, Nanquan, and Changquan. The composite score for all the seven movements of the FMS was recorded and then compared with the injury documentation. The majority of Wushu routine athletes (about 70%) were able to get three points from the in-line lunge, shoulder mobility, active straight leg raise and trunk stability push-up, which are related to the movement characteristics of Wushu routine. Thus, the FMS scores were relatively high in the most Wushu athletes as compared to previously published results in other sports [19], which highlights the need to identify sport-specific FMS score threshold for these athletes.

A lower FMS score is associated with an elevated injury risk [15]. We found that Wushu athletes who had FMS score less than 16 (sensitivity = 80%, specificity = 56%) had a higher odd of having some injury history (OR = 5.096), than those who had score over 16. Previous studies have highlighted that the lower scores in FMS testing (≤14) are often associated with a higher risk of sports injuries [23,24]. For example, Kiesel et al. found that professional male football players who had FMS score ≤14 had an eleven times greater risk of injury during the follow up season when compared to those who had a score over 14. However, they limited their data collection to “serious” injuries [14]. Garrison et al. reported that both female and male athletes presenting with FMS scores ≤14 experienced injuries 5.6 times more likely than those with scores>14 [25]. A similar association (OR = 3.8; 95%CI = 0.9–15.1) was also reported in a sample of 38 female collegiate athletes [26]. Cowen studied male and female firefighters whose mean baseline FMS score was 13, which was lower than in our current study [27]. However, it should be noted that the retrospective design could cause these differences than the prospective studies [25,26]. Shojaedin et al.and Letafatkar et al. indicated that an athlete has an approximately 4.7 times greater chance of suffering a lower extremity injury during a regular competitive season if they score less than 17 on the FMS, where both of they used the history of injury [28,29]. Accordingly, our results (Table 3) are well in line with the previous literature, such that Wushu routine athletes without injury history had higher FMS scores than those who had suffered injuries (17.7 ± 1.6 vs. 15.5 ± 3.1, *p* < 0.001).

Interestingly, the highest level Wushu routine athletes in the present study received lower scores in the FMS test, as compared to the lower level Wushu routine athletes. This suggests that a proper movement control is not developing purely by practicing the Wushu sport. In addition, the highest level Wushu routine athletes had the longest training history (on average 10.6 years), and thus a long-term exposure to potentially injurious events, which likely explains the higher occurrence of injuries. Because the longer training history was related with a lower FMS scores, injury prevention interventions can be needed especially in the group of highest-level Wushu athletes.

According to current results, Taiji athletes outperformed Nanquan athletes and Changquan athletes on average score on shoulder mobility, in-line lunge, and rotary stability. Additionally, Taiji athletes scored all test points of 18.29 ± 1.35(17.60–18.99). Changquan athletes outperformed Nanquan athletes on average score on in-line lunge, hurdle step and the rotary stability tests, while the Nanquan athletes were on average better on the trunk stability push-up. However, the average score of the rotary stability is only 1.77 ± 0.63(1.64–1.91). The rotary stability test was different from the other FMS tests in that few participants were able to obtain the maximum score of three, and most of participants got two points. This test demands trunk stability in the sagittal and transverse planes during asymmetric movement of the upper and lower limbs. The FMS training manual suggested that it is difficult to obtain a score of three (None of the athletes in the study scored a three point), but it is combined to capture elite performance [5,6]. It is questionable if this test serves a practical role in a screen for the general athletic population, and future study on the FMS method may need to improve and consider the combination of the different sports performance [18,19].

It should be noted that future studies need to consider the potential threshold scores for functional tests. Moreover, prospective studies that include detailed injury surveillance and a more robust injury definition are needed, in order to compare results across different studies [15]. While FMS can be used as a measuring tool and method to predict sports injury for Wushu athletes, the score of FMS test is limited to relate to the incidence of sports injury. One study found that persons who recorded pain during one of the seven subtests were more likely to sustain an injury [30]. This demonstrates that the FMS can be used beyond just the composite score to predict injuries. Low score of single sub-test and pain may also provide information for researchers to predict sports injuries.

One of the limitations of this study is the lack of the follow-up injury survey. Related follow-up injuries can provide more information for the feasibility of FMS scores for predicting sports injuries, and provide data on FMS recovery intervention training effectiveness. It is unknown if these results reflect the effect of low FMS score on injuries, or if the injury history resulted scoring lower in the FMS score. Future studies can consider tracking the prospective injury information during the competition season and provide training intervention to prevent the potential injury. We also recommend further prospective designs of study using the cut-off of 16 points to validate the accuracy of the FMS scores in the Wushu athletes.

## 5. Conclusions

This study found that the score of 16 in the FMS method was related to an increased number of injuries at the past training history of professional Wushu athletes. With the low cost and its simplicity, the FMS method can be used in the professional sports population for injuries evaluation. Future studies should utilize a prospective design to validate FMS with cut-off of 16 points in Wushu athletes.

## Figures and Tables

**Table 1:** Participants’ characteristics.

ALL (*n* = 84)	Mean ± SD (95% CI)
Age, years	15.1 ± 4.5 (14.1–16.1)
Height, cm	153.3 ± 15.3 (149.9–156.6)
Weight, kg	46 ± 15.2 (42.7–49.3)
Training year	6.2 ± 3.9 (5.4–7.1)
Female, %(*n*)	48.8 (41)
Training type, %(*n*)	
Taiji	20.2 (17)
Changquan	57.1 (48)
Nanquan	22.6 (19)
Training level, %(*n*)	
TOP	27.4 (23)
Q1	8.3 (7)
Q2	39.3 (33)
Q3	25 (21)

**Table 2:** The FMS scores for all participants.

Variable (*n* = 84)	Mean ± SD (95% CI)
Deep squat	2.17 ± 0.56 (2.05–2.29)
Hurdle step	2.32 ± 0.49 (2.21–2.43)
Lunge	2.61 ± 0.69 (2.46–2.76)
Shoulder mobility	2.65 ± 0.74 (2.49–2.81)
Active straight leg raise	2.95 ± 0.26 (2.89–3.01)
Trunk stability push-up	2.76 ± 0.79 (2.59–2.93)
Rotary stability	1.77 ± 0.63 (1.64–1.91)
Total scores	17.24 ± 2.2 (16.76–17.72)

**Table 3:** 2 × 2 contingency table indicating if an athletes’ FMS score was above or below the optimal cut-off point and if they had reported injury history.

	Injured	
	Yes	No
FMS™ Score ≤16	10	13
FMS™ Score >16	8	53

## Data Availability

The data presented in this study are available on request from the corresponding author.

## Supplementary Materials

The following are available online at https://www.mdpi.com/1660-4601/18/2/721/s1. Table S1: The detailed FMS scores of participants separately in different training levels (Top, Q1, Q2 and Q3) and training types (Taiji, Changquan and Nanquan). Figure S1: The frequency of FMS scores across the 7 tests (0–3 points) for different training levels (TOP, Q1, Q2 and Q3). Figure S2: The frequency of FMS scores across the 7 tests (0–3 points) for different training types (Taiji, Chuangquan and Nanquan).
