# Supplementary material for: Can the Functional Movement Screen Method Identify Previously Injured Wushu Athletes?
Source: Int J Environ Res Public Health. 2021 Jan 15;18(2):721. doi: 10.3390/ijerph18020721 (PMC7829906; doi:10.3390/ijerph18020721)
Supplement: Supplementary file 1 [file ijerph-18-00721-s001.pdf]

Supplementary Table S1. The detailed FMS scores of participants separately in different training levels (Top, Q1, Q2 and Q3) and training types (Taiji, Changquan and Nanquan).

**Variable Mean±SD (95%CI)**	**TOP (n=23)**	**Q1 (n=7)**	**Q2 (n=33)**	**Q3 (n=21)**	**Taiji (n=17)**	**Changquan (n=48)**	**Nanquan (n=19)**
Deep squat	2±0.52 (1.77-2.23)	1.86±0.38 (1.51-2.21)	2.33±0.54 (2.14-2.52)	2.19±0.6 (1.92-2.46)	2.24±0.56 (1.95-2.52)	2.17±0.55 (2-2.33)	2.11±0.56 (1.83-2.38)
Hurdle step	2.3±0.56 (2.06-2.55)	2.43±0.53 (1.93-2.92)	2.36±0.49 (2.19-2.54)	2.24±0.44 (2.04-2.44)	2.47±0.51 (2.21-2.74)	2.33±0.51 (2.18-2.48)	2.16±0.37 (1.98-2.34)
Lunge	2.48±0.9 (2.09-2.87)	2.57±0.53 (2.08-3.07)	2.7±0.64 (2.47-2.92)	2.62±0.59 (2.35-2.89)	2.76±0.56 (2.48-3.05)	2.6±0.7 (2.4-2.81)	2.47±0.77 (2.1-2.85)
Shoulder mobility	2.17±1.15 (1.67-2.67)	2.86±0.38 (2.51-3.21)	2.85±0.36 (2.72-2.98)	2.81±0.4 (2.63-2.99)	3±0 (3-3)	2.56±0.79 (2.33-2.79)	2.58±0.83 (2.18-2.98)
Active straight leg raise	3±0 (3-3)	3±0 (3-3)	2.94±0.24 (2.85-3.03)	2.9±0.44 (2.71-3.1)	3±0 (3-3)	2.96±0.2 (2.9-3.02)	2.89±0.45 (2.67-3.12)
Trunk stability push-up	2.48±1.16 (1.98-2.98)	2.57±1.13 (1.52-3.62)	2.91±0.52 (2.72-3.09)	2.9±0.3 (2.77-3.04)	2.94±0.24 (2.82-3.07)	2.63±1 (2.33-2.92)	2.95±0.22 (2.84-3.06)
Rotary stability	1.65±0.78 (1.32-1.99)	1.71±0.76 (1.02-2.41)	1.82±0.58 (1.61-2.03)	1.86±0.48 (1.64-2.07)	1.88±0.48 (1.63-2.13)	1.77±0.62 (1.59-1.95)	1.68±0.74 (1.32-2.05)
Total scores	16.09±2.91 (14.83-17.34)	17±2.16 (15-19)	17.91±1.81 (17.27-18.55)	17.52±1.33 (16.92-18.13)	18.29±1.35 (17.6-18.99)	17.02±2.51 (16.29-17.75)	16.84±1.67 (16.03-17.65)

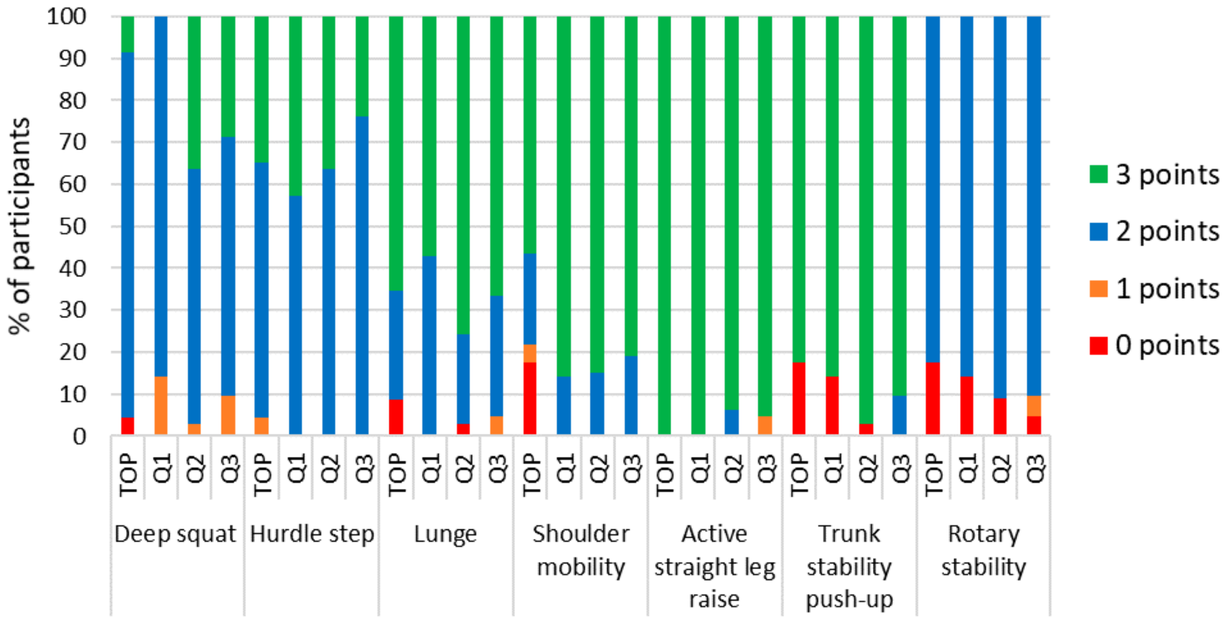

Supplementary Figure S1. The frequency of FMS scores across the 7 tests (0–3 points) for different training levels (TOP, Q1, Q2 and Q3)

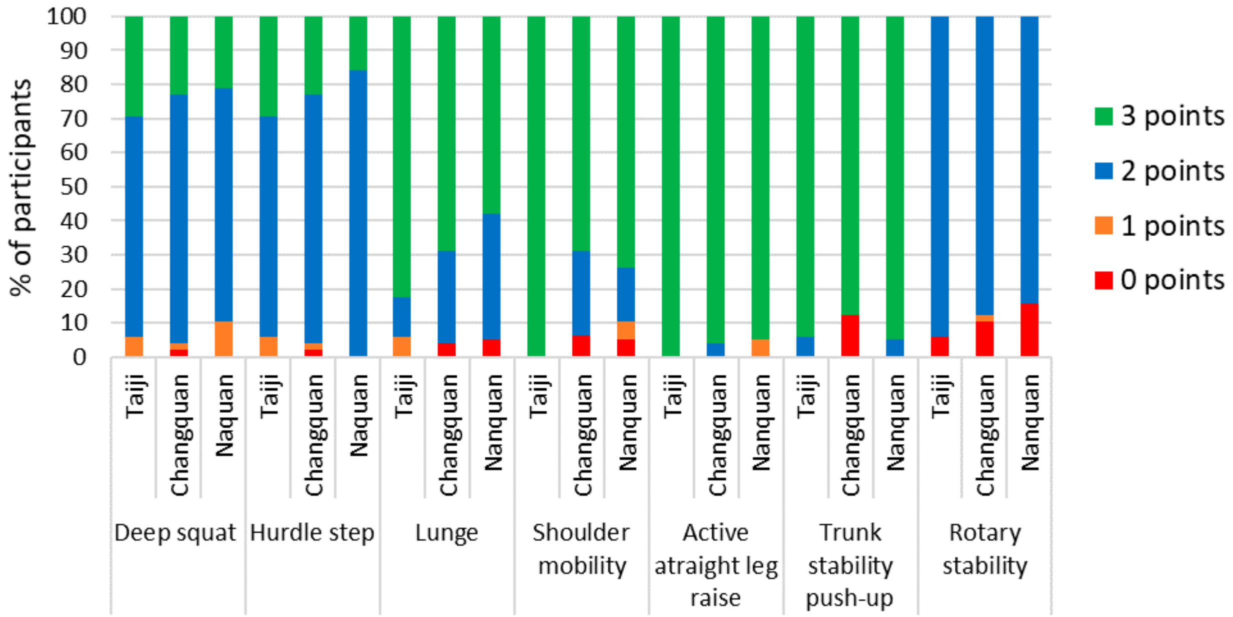

Supplementary Figure S2. The frequency of FMS scores across the 7 tests (0–3 points) for different training types (Taiji, Chuangquan and Nanquan)
